# Speech Disfluencies in Consecutive Interpreting by Student Interpreters: The Role of Language Proficiency, Working Memory, and Anxiety

**DOI:** 10.3389/fpsyg.2022.881778

**Published:** 2022-05-27

**Authors:** Nan Zhao

**Affiliations:** Department of Translation, Interpreting and Intercultural Studies, Hong Kong Baptist University, Kowloon, Hong Kong SAR, China

**Keywords:** interpreting, language proficiency, working memory, anxiety, disfluency

## Abstract

Growing research has revealed that interpreters’ individual cognitive differences impact interpreting. In this article, I examined how an interpreter’s language proficiency, working memory, and anxiety level impact speech disfluencies in target language delivery. Fifty-three student interpreters took part in three cognitive tests, respectively, of their proficiency in English (their non-native language), working memory, and anxiety level. Then they consecutively interpreted an English speech into Mandarin (their native language); their target language output was coded for different types of disfluencies (pauses, fillers, repetitions, and articulatory disfluency). It was found that anxiety level, but not language proficiency and working memory, impacted the occurrence of disfluencies in general. In particular, more anxious interpreters tended to have more fillers, such as *er* and *um*, and more repetitions of words and phrases. I discuss these findings in terms of how anxiety may impact the cognitive processes of interpreting and how to reduce student interpreters’ anxiety level in interpreting teaching and learning.

## Introduction

Interpreters translate from a source language to a target language. Such a task is often delivered under time pressure, in front of an audience, and requires multitasking. For instance, in consecutive interpreting, a speaker delivers a segment of speech (varying from one to a dozen sentences) and the interpreter needs to quickly transcode the source language (e.g., words, syntactic structure) into the target language, which they keep in their working memory (or on a note); then, when the speaker pauses, they output the target language as fluently and accurately as possible to an audience. Thus, to successfully accomplish an interpreting task, the interpreter needs to, among other things, be fully proficient in both the source and target language, actively keep a large amount of linguistic information in their working memory, and overcome the anxiety and stress of public speaking.

In this article, I focus on one important aspect of target language delivery, namely speech disfluencies in the target language. I examine how speech disfluencies vary as a function of the interpreter’s cognitive traits: language proficiency, working memory, and anxiety. Below, I first review how these different cognitive traits may impact interpreting and then review speech disfluencies in interpreting, before reporting my own study.

### Language Proficiency in Interpreting

As interpreters often interpret between a native language and a non-native language, proficiency in the non-native language is critical for interpreting. Blasco Mayor (2015) showed that student interpreters’ listening comprehension ability predicts their interpreting performance. She argued that it’s important to train students’ listening skills in interpreting teaching and learning. Jiménez Ivars et al. (2014) showed that both interpreting performance and self-efficacy in student interpreters increased as a function of their non-native language proficiency. Christoffels et al. (2003) also showed that the speed with which interpreters retrieve translation equivalents between languages and the speed with which they name pictures are correlated with their interpreting performance, again highlighting the role of language proficiency in interpreting. Indeed, there is evidence that interpreting training often improves student interpreters’ language skills compared to non-interpreting bilingual controls (Tzou et al., 2011).

### Working Memory in Interpreting

Working memory has been shown to play a critical role in many aspects of language processing, including language comprehension (e.g., Daneman and Carpenter, 1983; Waters and Caplan, 2005) and language production (e.g., Belke, 2008; Martin and Slevc, 2014). As interpreting involves both the comprehension of the source language and the production of the target language, it is no surprise that working memory has long been assumed to likewise play a critical role in interpreting models (Gerver, 1975; Moser, 1978; Darò and Fabbro, 1994). There is also empirical evidence for the role of working memory in interpreting. First, it has been shown that trained interpreters outperformed bilingual controls on working memory tests (Christoffels et al., 2006; Hodáková, 2009; Tzou et al., 2011), suggesting that cognitive resources (i.e., working memory) are a critical sub-capacity for interpreting. However, other studies have not found a reliable difference in working memory capacities between interpreters and bilinguals controls (Liu et al., 2004; Köpke and Nespoulous, 2006).

Instead of comparing interpreters with bilingual controls, other studies have investigated whether interpreting performance relates to an interpreter’s working memory capacity. Timarová (2008) showed that some working memory functions (especially the capacity to inhibit irrelevant information) correlate with simultaneous interpreting performance. Christoffels et al. (2003) showed that working memory makes a contribution to interpreting skills independent of an interpreter’s language proficiency. There is also evidence that a sign interpreting performance correlates with working memory span (Van Dijk et al., 2012). These findings thus point to a positive correlation between working memory and interpreting performance.

### Anxiety in Interpreting

It goes without saying that interpreting is a very stressful activity because it involves performing a series of complex cognitive and psychomotor for an audience, whether in public or private. Students training to become interpreters have to overcome anxiety and stress about having to speak (interpret) in public. Indeed, researchers have long considered the capacity to control anxiety and stress as an important requisite for a good interpreter (Cooper et al., 1982; Moser-Mercer, 1985; Longley, 1989; Klonowicz, 1994; Gile, 1995; Moser-Mercer et al., 1998) and a predictor of an interpreter’s competence (Alexieva, 1997; Dong et al., 2013a). Some researchers have proposed to take the capacity to control anxiety and stress while interpreting into account in interpreting entrance exams (e.g., Moser-Mercer, 1985).

Empirical research has emphasized professional interpreters at work, focusing mainly on physiological responses to stress during interpreting: cardiovascular activity (Klonowicz, 1994), causes of anxiety and stress (Cooper et al., 1982), and chemical and physiological analysis (Moser-Mercer et al., 1998). There is now good evidence that interpreting leads to anxiety and stress for the interpreter (e.g., Cooper et al., 1982; Kurz, 1997, 2003). Thus, it is likely that interpreters, especially inexperienced ones like student interpreters, may experience a high level of anxiety when delivering target language, resulting in speech disfluencies (e.g., Cho and Roger, 2010). Indeed, people tend to stutter more when they are anxious (Craig, 1990; Menzies et al., 1999; Messenger et al., 2004). Because stuttering is an extreme example of disfluency, it is likely that anxiety may also lead to disfluencies in speech. Indeed, anxiety has long been associated with foreign language speaking (e.g., MacIntyre and Noels, 1996), leading to disfluencies in foreign language speech (Arnaiz and Pérez-Luzardo, 2014) and public speaking (Andrade and Williams, 2009).

### Speech Disfluencies in Language Production and in Interpreting

Disfluencies are generally described as interruptions of the execution of a speech plan (Postma et al., 1990). As a form of language production, target language output is also filled with speech disfluencies. Some research has looked into speech disfluencies in simultaneous interpreting. Pöchhacker (1995) examined speech repairs (e.g., false starts, lexical blends, and syntactic blends) in conference source speech and corresponding simultaneous interpreting output (between English and German in both directions). More speech repairs were found in the target language output than in the source language output. There were more simple errors and false starts in the output of speakers, whereas in the output of interpreters, the most frequent disfluencies were lexical and structural blends.

Mead (2000) examined the control of pauses when students interpreted into their native or non-native language. Using Gósy’s (2007) taxonomy, a series of papers were published on speech disfluencies in the output of simultaneous interpreters working in Hungarian.

Tissi (2000) attempted to come up with a simultaneous interpreting–specific taxonomy of disfluencies and at the same time stressed the communicative value and the strategic use of disfluencies in interpretation. She focused on silent pauses (the two subcategories being grammatical and/or communicative pauses and non-grammatical pauses) and disfluencies (including fillers such as vocalized hesitations, vowel and consonant lengthenings, and interruptions such as repeats, restructuring and false starts). Tissi found large individual variations, and argued that no clear trends can be identified and that the influence of the source speech is not as direct as one would assume. She also found that vowel and consonant lengthenings are much more numerous in the target speech, and false starts occur only in the target speech. She also noticed the communicative, sometimes even strategic, use of disfluencies by the interpreter (e.g., silent or filled pauses before a correction), lengthenings of the tonic vowel, and retrospective repeats.

In this article, following the psycholinguistic literature (e.g., Postma et al., 1990; Fox Tree, 1993), I propose that interpreting disfluencies mainly include pauses, fillers, repetitions, and articulatory disfluencies, among others (see Table 1 below)^1^.

**TABLE 1:** Classification of disfluencies in interpreting, with examples.

Type of disfluency	Definition and example
Pause (DP)	A silence inside a clause
	E.g., *And companies like China Mobile*… *同样的像中*<*DP*>*国移动这样的公司*……
Filler (DF)	The use of speech signals such as “uh,” “mm,” etc., to fill a pause
	E.g., *I need 100 million units*… *需要呃*……<*DF*>, *呃*……<*DF*>*一千亿的, 呃*……*订购 。*
Repetition (DRe)	The repetition of a single Chinese morpheme, a whole word or a phrase (in order to buy time for subsequent lexical access)
	E.g., *I watch all the time, students made perfectly beautiful programs 我曾经, 曾经*<*DRe*>*见过很多学生, 他们可以做出来很好, 的*<*DRe*>*他们可以做出来很好的*
Articulatory disfluency (DAr)	The stuttering of a morpheme within a word
	E.g., 1: *And the reason I’m focused on children is because*… *程序。*<*DAr*>, *我之*…… E.g., 2: *We want to make*… *之所以去进行这一个项目的原因*<*DAr*>, *我*<*DAr*>, *我*……
Other disfluency	Unidentified disfluencies that don’t fit into the above categories

*In the examples, the English text is the source language and the Chinese text is the target language. Letter strings in brackets (e.g., <DP>) are codes for different disfluency types and are used here to indicate the position of the disfluency.*

Speech is often disrupted by (silent) pauses and fillers (filled pauses). Pauses are a period of silence in the middle of an utterance, often caused by speech-planning problems. But in interpreting (as in conversation), a pause in speech may be ambiguous to the speaker and the audience, who may take it to signal the end of the interpreting. Thus, interpreters (as speakers) tend to have filled pauses (or fillers) during speech. Fillers specifically refer to *uh* and *um* (and equivalents in other languages), which are very common in speech production (e.g., Clark and Fox Tree, 2002), and have received much attention in recent years. According to Clark and Fox Tree, *uh* and *um* in English are signals that allow the speaker to keep the floor during conversation so that he/she will have more time for language planning (e.g., searching for words or framing the message). Thus, I also assume that interpreters use fillers strategically to hold the floor during interpreting, especially when there is a speech planning problem.

Repetitions occur when an interpreter or a speaker repeats a word or words without any grammatical or apparent semantic purposes (e.g., *she*… *she likes it*). In natural speech, speakers tend to repeat function words such as articles (e.g., *the, a*), prepositions (e.g., *of*), and auxiliaries (e.g., *do*) more often than content words such as nouns and verbs (e.g., Fox and Jasperson, 1995), probably because function words tend to begin a phrase (e.g., Clark and Wasow, 1998). Furthermore, another type of word that is often repeated is pronouns, especially when they begin a phrase (Clark and Wasow, 1998). For instance, it was shown that the possessive *her* (e.g., *her son*) was repeated more frequently than the accusative pronoun *her* (e.g., *love her*), despite the fact that they have the same form (Clark and Wasow, 1998).

Finally, speech can be disrupted when the speaker experiences articulatory disfluencies such as stuttering. Articulatory disfluencies can be seen in non-stuttering interpreters/speakers, often manifesting as difficulty producing a syllable in the middle of a word (e.g., *sec..secondary*). Articulatory disfluencies thus occur as a result of difficulties during speech programming rather than intentionally repeating a word (as a repetition).

### The Current Study

The quality of interpreting depends on, among other things, two important criteria: accurate delivery of content in the source language and fluent delivery of the target language (e.g., Zhao and Dong, 2013). The former can be reflected in the likelihood of erroneous interpreting (see Zhao et al., 2021) and the latter can be reflected in the (dis)fluency of interpreting output. In this article, I focus on speech disfluencies in interpreting. In particular, I examine how different types of interpreting disfluencies relate to a student interpreter’s cognitive traits, in particular, to a student interpreter’s language proficiency, working memory span, and anxiety.

As I reviewed above, there is much evidence that language proficiency, working memory, and anxiety impact how well the interpreter conducts interpreting. As a specialized form of bilingual language processing, consecutive interpreting involves both the comprehension of a source language and the production of a target language. Therefore, it is critical that interpreters have sufficient proficiency in both the source and target language (Blasco Mayor, 2015). In addition, consecutive interpreting requires the storage of much source language information in working memory before it can be delivered in the target language; hence, working memory capacity is also shown to be critical in interpreting performance (e.g., Christoffels et al., 2006). Finally, consecutive interpreting is a form of public speaking where interpreters convey a message to an audience, often in a formal setting; therefore, the capacity to control anxiety has traditionally been considered one of the requisites for interpreting (e.g., Moser-Mercer et al., 1998) and a predictor of interpreting competence (e.g., Alexieva, 1997).

To examine how language proficiency, working memory and anxiety may impact interpreting disfluencies, I conducted an experiment where 53 student interpreters consecutively interpreted an English speech into Chinese. I also measured their proficiency in English (the source language), working memory span in English listening, working memory span in Chinese speaking, and their general anxiety about public speaking. Interpreting output of the student interpreters was coded for disfluencies (see Table 1 for a taxonomy of interpreting disfluencies). I then used regression analyses to examine the relationship between student interpreters’ cognitive traits and interpreting disfluencies.

## Materials and Methods

### Participants

Fifty-three fourth-year college students (45 females and 8 males; the imbalance of gender reflects female dominance in interpreting students in China) majoring in interpreting and translation participated in the consecutive interpreting test in a session of their interpreting module. These students all spoke Mandarin Chinese as their first language and had learned English as a second language since primary school. In addition, they all majored in English in college and had used English in both their courses and daily life. Thus, they were all unbalanced Chinese-English bilinguals who were proficient in English. All these participants trained in English language in the first 2 years of their university education and started to train in interpreting from the 3rd year onward (i.e., they had already had 1 year of interpreting training at the time they participated in this study).

### The Language Proficiency Test

All 53 participants further took part in a language proficiency test. I developed our test on the basis of the Test for English Majors Band 8, which is a national official test of English language proficiency for English majors in the fourth BA year (such as our participants). As some of the test items were not relevant to language proficiency (e.g., test items on linguistics and English literature), I selected only test items that were related to proficiency in real language usage; these included the reading comprehension part, the listening comprehension part, and the writing composition part (see Supplementary Appendix 1 for a description of the test items). The total score was 56. In the test, after test papers and answer sheets were distributed to the participants, they began the test with the listening comprehension part, followed by the reading comprehension part, and then by the writing composition part.

### The Working Memory Test

The working memory test was adapted from the paradigm developed in Mizera (2006), in which participants memorized a list of Chinese words [e.g., 数学 (math), 现代 (modern), 面积 (area)] and then made a sentence for each word. The materials were 100 two-character Chinese words; all were high-frequency words according to the Modern Chinese Word Frequency Dictionary. There were 5 sets of test items, respectively, with 2, 3, 4, 5, and 6 memory words in a trial. There were 5 trials in each set, with a total of 25 trials. In each trial, participants first read the words one by one on a computer screen, with each word being presented for 1 s. After the presentations, a cue sentence appeared on the screen asking participants to make up a sentence for each of the words presented. Participants pressed the spacebar and made up the sentences. All responses were digitally recorded. Trials were randomly presented. There was a practice session with two trials, one with 2 memory words and one with 3. The score for the test was the proportion of words (out of 100) with which a grammatical sentence was composed.

### The Anxiety Questionnaire

Note that anxiety in interpreting may be a multifaceted factor that consists of a student interpreter’s general daily anxiety (e.g., when dealing with people and when doing a job) and his/her anxiety about interpreting (e.g., not being very good at English or having a poor memory). In order to exclude language-related and memory-related factors (which were covered by language proficiency and working memory tests already), I decided to use a scale developed in Zhang and Schwarzer (1995) and translated into Chinese by Dong et al. (2013b; see Supplementary Appendix 2 for sample questions). The scale consisted of two parts. Part 1 tested self-efficacy anxiety (i.e., the anxiety one feels regarding whether he can do a particular task) and Part 2 tested state-trait anxiety (anxiety level as a personal characteristic). An answer was scored 1, 2, 3, or 4 points depending on the response, and a person’s total score for anxiety was the sum of all the points in the 30 test items.

### The Interpreting Test

The source language (English) speech was adapted from a real international conference speech on computer technology (see Supplementary Appendix 3). The original speech lasted for about 10 min, with a speech rate about 180 words per minute; such a speech rate is deemed to the most natural and pleasing speed for broadcasting (Boyd, 2003). The speech was delivered in a standard American accent. The speech was segmented to make it suitable for consecutive interpreting. In line with the common practice of the China Aptitude Test for Translators and Interpreters (CATTI) for consecutive interpreting Level II, following each segment of speech (2–5 sentences in length) was a pause that lasted for about 1.5 times the duration of the preceding segment, where student interpreters provided their interpreting.

The interpreting test was conducted by a teacher in a multimedia lab where participants had their interpreting classes. Participants sat in front of a computer with their headphones. The teacher gave verbal instructions regarding the interpreting test. In the test, participants heard the speech segment by segment, during which note-taking was allowed. At the end of each segment, participants heard an audio signal “ding” as a cue to start their interpreting. Participants’ interpreting was individually recorded. The test lasted about 25 min.

I then invited two experienced professional interpreters to rate student interpreters’ performance. Both raters had worked as professional consecutive and simultaneous interpreters for over 8 years and taught interpreting courses on BA and MA level at a university for 6 years by the time of rating. They took part in a rater training session on the rating scale before conducting the rating. They then rated two interpreting recordings (not part of the recordings in the current study) using the scale. For rating discrepancies, they discussed and reached a common ground. After this, they separately rated each student’s interpreting according to the rating scale (with a full score of 100; Zhao and Dong, 2013). I computed an average score for each participant. Then the recordings of interpreting were transcribed. On the basis of the transcriptions, disfluencies were coded according to the taxonomy I reviewed above (see also Table 1 for examples).

## Results

I first tested how interpreting score varied as a function of the three cognitive factors (see Supplementary Material for the data). Participants’ interpreting scores increased as a function of their language proficiency (β = 1.07, SE = 0.33, *t* = 3.4, *p* = 0.002), increased as a function of their working memory (β = 0.36, SE = 0.10, *t* = 3.40, *p* = 0.002), and decreased as a function of their anxiety level (β = −0.22, SE = 0.08, *t* = −2.66, *p* = 0.011). I also found a significant correlation between a participant’s interpreting score and their total disfluency rate (*r* = −0.40, *t* = −3.11, *p* = 0.004): participants who had a higher overall disfluency rate tended to do more poorly in their interpreting performance.

**FIGURE 1:**
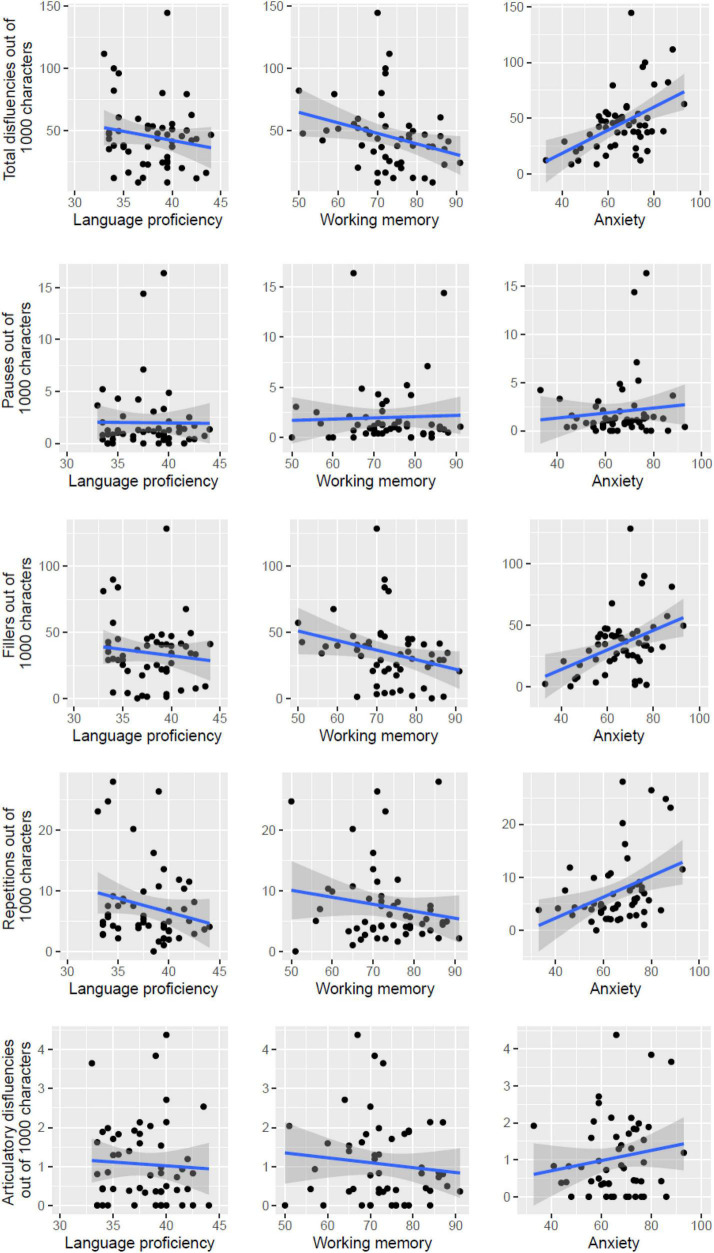
Total disfluencies, pauses, fillers, repetitions, and articulatory disfluencies as a function of language proficiency, working memory, and anxiety.

I next examined the occurrence of disfluencies and how they might be impacted by cognitive factors. In general, there were about 45 disfluencies out of 1,000 morphemes/characters in the target language output. Among the different disfluency types, the most common one is fillers, followed by repetitions. Pauses and articulatory disfluencies were rare (see Table 2). I conducted regression analyses on the rate of total disfluencies (i.e., number of disfluencies out of 1,000 characters in the output speech), using language proficiency, working memory and anxiety as predictors. As shown in Table 3; see also Figure 1, there is no significant effect. There is a marginally significant effect of working memory, with a trend of disfluencies decreasing as a function of working memory. There is a significant effect of anxiety, with increasing disfluencies as a function of participants’ anxiety level.

**TABLE 2:** Descriptive statistics of different disfluency rates (out of 1,000 characters in target output).

Type	Range	Mean	SD
Total disfluencies	8.6–144.4	45.3	26.6
Pauses	0–16.4	2.0	3.1
Fillers	0.4–128.4	34.5	24.2
Repetitions	0–28	7.4	6.5
Articulatory disfluencies	0–4.4	1.1	1.1

**TABLE 3:** Different types of disfluencies as a function of the cognitive factors (significant *p*-values in bold).

	*Estimate*	*SE*	*t*	*p*
**Total disfluencies**				
(Intercept)	50.62	60.40	0.84	0.406
Language proficiency	−0.53	1.12	−0.47	0.639
Working memory	−0.62	0.35	−1.75	0.086
Anxiety	0.92	0.28	3.25	**0.002**
**Pauses**				
(Intercept)	−2.48	8.13	−0.31	0.762
Language proficiency	0.03	0.15	0.17	0.866
Working memory	0.02	0.05	0.42	0.674
Anxiety	0.03	0.04	0.81	0.422
**Fillers**				
(Intercept)	37.66	57.49	0.66	0.516
Language proficiency	−0.23	1.06	−0.22	0.829
Working memory	−0.55	0.34	−1.63	0.109
Anxiety	0.70	0.27	2.60	**0.012**
**Repetitions**				
(Intercept)	12.74	15.88	0.80	0.426
Language proficiency	−0.28	0.29	−0.97	0.339
Working memory	−0.08	0.09	−0.83	0.413
Anxiety	0.17	0.07	2.27	**0.028**
**Articulatory disfluencies**				
(Intercept)	1.27	2.76	0.46	0.649
Language proficiency	−0.01	0.05	−0.14	0.887
Working memory	−0.01	0.02	−0.60	0.551
Anxiety	0.01	0.01	0.92	0.361

*significant p-values in bold.*

Finally, I looked at how different types of disfluencies varied as a function of the three cognitive factors. Pauses did not vary as a function of any cognitive factor. Fillers did not vary as a function of language proficiency and working memory, but increased as a function of anxiety. Repetitions did not vary as a function of language proficiency and working memory, but increased as a function of anxiety. Finally, articulatory disfluencies did not vary as a function of any cognitive factor. It should, however, be noted that the occurrences of pauses and articulatory disfluencies were rare and the lack of cognitive influences on these disfluencies could be due to a floor effect.

## Discussion

In this study, I explored how a student interpreter’s cognitive traits, namely language proficiency, working memory and anxiety level, impacted speech disfluencies in target language delivery. Student interpreters were judged as worse in interpreting performance if they produced more disfluencies. Importantly, I showed that the occurrence of disfluencies is influenced by a student interpreter’s anxiety level but not their language proficiency or working memory. In particular, student interpreters with higher anxiety tended to have more fillers and more repetitions in their interpreting output. My findings of the anxiety effects are thus in line with previous theorizing that anxiety control is an important part of interpreting ability (Cooper et al., 1982; Moser-Mercer, 1985; Klonowicz, 1994; Gile, 1995; Alexieva, 1997; Moser-Mercer et al., 1998).

But how does anxiety affect the fluency of target language delivery in interpreting? According to the attentional control theory of anxiety (Eysenck et al., 2007), an influential theory that specifically addresses how anxiety affects cognitive performance (which includes interpreting), anxiety increases stimulus-driven attention (i.e., automatic attention to salient things, e.g., a loud sound) but decreases goal-driven attention (i.e., attention needed to complete a goal, e.g., interpreting a speech). More specifically, when an individual feels anxious, he/she attends more to salient properties in the surrounding environment; when the properties are not goal-related (e.g., a cough from the audience when an interpreter is working), the individual is easily distracted, thus leading to processing difficulties (e.g., at finding an appropriate translation word) and in turn to disfluencies (e.g., fillers).

According to the attentional control theory, an anxious individual is impaired in his/her cognitive functions that are necessary for completing a goal. These cognitive functions include inhibition, shifting, and updating (see also Miyake et al., 2000). Inhibition is a cognitive process whereby an individual is less likely to respond to things (e.g., responding to a goal-irrelevant in the audience during interpreting). Shifting is needed to allocate cognitive resources among different sub-tasks (e.g., listening to the source speech while retrieving target language expressions) when a cognitive performance requires multitasking. Finally, updating is a process that helps to update and monitor working memory representations (e.g., semantic representations retrieved from the comprehension of the source speech). Both attention and executive functions (e.g., inhibition and updating of information) are necessary in language processing and thus interpreting. For example, in language production such as interpreting, attentional resources are necessary to monitor whether a produced speech contains errors, and executive functions such as updating are necessary to integrate the message from a new sentence into the context to build a coherent model of the topic being comprehended. Thus, it is expected that an interpreter’s anxiety has an all-round impact on interpreting (e.g., comprehension of source language, content delivery in the target language), not just disfluencies.

Given the crucial role of anxiety in interpreting, helping student interpreters to become less anxious (especially in public) should be an important component of the interpreting training curriculum. To do this, it is crucial that we understand the anxiety level of each student interpreter. We can then build an anxiety profile for each student by regularly testing their anxiety level (e.g., Dong et al., 2013b). For anxious students, more opportunities should be offered for them to speak in public.

In summary, we showed that speech disfluencies, especially fillers and repetitions, tended to increase as a function of a student interpreter’s anxiety level. Given that interpretation is expected to be as fluent as possible, the finding suggests that interpreting teaching and learning should place more emphasis on reducing student interpreters’ anxiety, especially in public speaking.

## Data Availability Statement

The datasets presented in this study can be found in online repositories. The names of the repository/repositories and accession number(s) can be found in the article/Supplementary Material.

## Ethics Statement

The studies involving human participants were reviewed and approved by the Guangdong University of Foreign Studies. The patients/participants provided their written informed consent to participate in this study.

## Author Contributions

The author confirms being the sole contributor of this work and has approved it for publication.

## Conflict of Interest

The author declares that the research was conducted in the absence of any commercial or financial relationships that could be construed as a potential conflict of interest.

## Publisher’s Note

All claims expressed in this article are solely those of the authors and do not necessarily represent those of their affiliated organizations, or those of the publisher, the editors and the reviewers. Any product that may be evaluated in this article, or claim that may be made by its manufacturer, is not guaranteed or endorsed by the publisher.

## Footnotes

1 Arguably speech repair is also a form of disfluency. However, I did not include this in the current study because speech repair is better treated as a form of speech monitoring (e.g., Levelt, 1983) and is thus beyond the scope of the current study. Interested readers can refer to Zhao (2015).
