# Supplementary material for: Speech Disfluencies in Consecutive Interpreting by Student Interpreters: The Role of Language Proficiency, Working Memory, and Anxiety
Source: Front Psychol. 2022 May 27;13:881778. doi: 10.3389/fpsyg.2022.881778 (PMC9197251; doi:10.3389/fpsyg.2022.881778)
Supplement: Supplementary file 1 [file Data_Sheet_1.docx]

**Appendix 1****.** Composition of the language proficiency test. The test had three sections (listening comprehension, reading comprehension and writing); some of these sections had sub-sections. The time information in the table is about the average time that was needed to complete each sub-section.

| Section | Sub-section | Content | Score | Time |
| --- | --- | --- | --- | --- |
| Listening comprehension | Note-taking and gap filling | 1 passage with 10 gaps to be filled | 10 | 15 min |
|  | Interview | One spoken passage with 10 questions | 10 | 5 min |
| Reading comprehension | Reading in depth | 2 written passages, each with 5 comprehension questions (10 questions in total) | 10 | 15 min |
|  | Reading and scanning | 4 written passages with 6 questions in total | 6 | 5 min |
| Writing |  | Write an essay of about 400 words on “one major problem in the process of urbanization” | 20 | 45 min |
