# Supplementary material for: Speech Disfluencies in Consecutive Interpreting by Student Interpreters: The Role of Language Proficiency, Working Memory, and Anxiety
Source: Front Psychol. 2022 May 27;13:881778. doi: 10.3389/fpsyg.2022.881778 (PMC9197251; doi:10.3389/fpsyg.2022.881778)
Supplement: Supplementary file 2 [file Data_Sheet_2.docx]

**Appendix 2.** Sample items from the anxiety questionnaire. In the questionnaire, the choices ①, ②, ③, and ④ respectively mean that the statement is “barely true”, “sometimes true”, “often true” and “always true”.

| Sample questions from Part 1  1.如果我尽力去做的话，我总是能够解决问题的。① ② ③ ④  2.即使别人反对我，我仍有办法取得我所要的。　① ② ③ ④  3.对我来说，坚持理想和达成目标是轻而易举的。① ② ③ ④  4.我自信能有效地应付任何突如其来的事情。 ① ② ③ ④  Sample questions from Part 2  1. 我感到愉快。 ① ② ③ ④  2. 我感到紧张不安。 ① ② ③ ④  3. 我对自己感到满意。 ① ② ③ ④  4. 我希望自己能够像别人一样开心。 ① ② ③ ④ |
| --- |
