# Supplementary material for: Speech Disfluencies in Consecutive Interpreting by Student Interpreters: The Role of Language Proficiency, Working Memory, and Anxiety
Source: Front Psychol. 2022 May 27;13:881778. doi: 10.3389/fpsyg.2022.881778 (PMC9197251; doi:10.3389/fpsyg.2022.881778)
Supplement: Supplementary file 3 [file Data_Sheet_3.docx]

**Appendix 3**. The adapted conference speech that served as the source language speech. The speech was segmented into different sections for consecutive interpreting; the segmentations are indicated by //.

*Thank you very much!*

*I have been… constantly telling, over the past 20 years, ministers of… telecommunications when I meet them, including the minister of telecommunications in China. And I said you know, …you don’t know this, but you are actually the minister of education.****//*** *And in the same way I think companies like China Mobile will in fact impact education in China more than any other… phenomenon… in China, because communications is education.* ***//***

*I wanna talk about simplicity for a moment. …What’s happened… is in my opinion …that we are as a world at a break point.* ***//*** *Now imagine that you are in an industry where in 18 months from now it will cost you …half to make what you are making today. When you are in that kind of industry, you certainly do not want …to let the cost of your products drop to half in 18 months. So what does Motorola, Intel or Lenovo, what do, what do these companies do? What they do is they add features.* ***//*** *And hopefully a year and half from now the additional features …will be sufficient that you will at least pay as much as, maybe a little more, for your next generation device. But what has happened is that these devices start getting fatter and fatter and fatter.* ***//***

*In the meanwhile we are doing the same thing with laptops. My laptop today which is the highest end laptop you can buy on the market runs slower than it did ten years ago. It is less reliable than it was 10 years ago. It crashes more than it did 10 years ago. What’s going on? It’s time to rethink these even though it’s human nature.* ***//*** *I watch all the time students write perfectly beautiful programs and demonstrate them at the beginning of a thesis or a project. And then they started adding features and features and features and features. We do this and we add that and we embellish this and we embellish that, and these things get fatter and fatter and fatter, whereas the simplicity and beauty of the program was very often much much better just in the very beginning.* ***//***

*Now I’m gonna use … the rest of my time to talk about One-Laptop-Per-Child. But think of it not as a laptop project. The laptop is almost irrelevant. It’s an education project.* ***//*** *Because this was the first project that really had laptops at scale, eh, in a very remote village, in fact in a village that has no electricity, no telephone, er, no television. And[Ə] there are four other villages involved, two of which have no road.* ***//***

*It breaks my heart to go into an African village where you maybe even can’t drive there. You have to walk the last few hundreds yards and you see a school. Usually a pretty ratty school with some metal on the roof and broken windows if there were ever windows at all. And you, 咳, you go in and you see a few u[~]sed computers driven by, er, a generator that’s out back probably making a lot of noise.* ***//*** *And I watch the 7, 8 and 9 year-olds being tau[~]ght WORD, EXCEL and PowerPoint. That is criminal. These are not office workers. They are not going into the job market. These kids should be making things, they should be sharing(笑) things, they should be accessing in the internet. They don’t need to use EXCEL and PowerPoint.* ***// “****Computer literacy” is the wrong concept. And I spend so much time trying to get heads of state to stop thinking about “computer literacy”. That is rubbish. What kids have to do is to learn about learning.* ***//*** *And one of the things we’ve done, in One-Laptop-par, per-Child is to make sure there are so many computer programming environments in that laptop that kids around the world will be writing programs again. And the kids in Ethiopia today, trust me, are writing some really fantastic computer programme as well as being on the internet.//*

*So in this picture …you see the children with their laptops… that were shipped to them. And after three and half years only one laptop broke. Whereas if we put laptops in the back of a classroom, in the United States at least, and let (s), (s), students check them out for a science class and put them back at the end of a class, those laptops are broken…… within less than three months. What’s the difference?* ***//*** *Difference’s simple. These kids sleep with their laptops. The little boys get their sisters to make… bags for them. They polish them. There …is nothing in their lives that is more important than their laptops and trust me they don’t break because they are highly highly protected. They are not government property, they are not school property, they belong to the kids.* ***//***

*And the reason I’m focused on children is because of those 1.2 billion children 500 million will not see anything… if we just use normal market forces. There is no reason for a company to go in that village. There’s nothing there! There’s no market.* ***//*** *Companies like China Mobile are covering the whole country, so they are like the lead[~] forces and very very important to this whole process. Because they do go to villages like that and so we think that the telecommunications is education is also coming from the fact that it is the wireless …carriers that are leading the effort and going into these places for a combination of business reasons as well as regulatory …reasons. …So when I looked at the picture I said what can we do to scale it?* ***//***

*Most projects that have to do with computers and education and connecting kids in remote places have been small projects. And when I started this I went to some of the display manufacturers and I said: You know I need a display that doesn’t have to be very bright, can have a few pixels missing, emm, it doesn’t have to be too big… but… it has to be very inexpensive.* ***//*** *The chairman of one of these companies …said to me: Well you know Nicholas, our corporate strategy is the opposite. We want to make big displays, 0 defects, perfect color, very bright, large. So everything in our corporate strategy is the opposite, so we can’t help you.* ***//*** *And I said: well that’s a shame, because I need 100 million units a year. He said: well, let me think about that. Maybe we can change our corporate strategy. They not only changed their corporate sta, strategy but a year ago they spent $1 billion building a new fab for this display.* ***//*** *And what’s important about that is the scale changes strategy. And that’s really very very important. In fact no body would be interested in One-Laptop-Per-Child if it was a small scale phenomenon.* ***//***

*And if you are interested in more information, please go to “laptop.org” and[Ə] with that thank you very very much.*
